# Preliminary results of a clinical study to evaluate the performance and safety of swallowing in critical patients with COVID-19

**DOI:** 10.6061/clinics/2020/e2021

**Published:** 2020-06-08

**Authors:** Maíra Santilli de Lima, Fernanda Chiarion Sassi, Gisele C. Medeiros, Ana Paula Ritto, Claudia Regina Furquim de Andrade

**Affiliations:** I Divisao de Fonoaudiologia, Hospital das Clinicas HCFMUSP, Faculdade de Medicina, Universidade de Sao Paulo, Sao Paulo, SP, BR; II Departamento de Fisioterapia, Fonoaudiologia e Terapia Ocupacional, Faculdade de Medicina FMUSP, Universidade de Sao Paulo, Sao Paulo, SP, BR

Coronavirus disease (Covid-19) is a viral respiratory illness (1). Whereas most infected individuals are asymptomatic or experience mild symptoms (*e.g.*, fever, cough, shortness of breath, and myalgia), 10-20% of patients develop acute respiratory distress syndrome (1). Intensive care unit (ICU) length of stay is relatively long, and the need for prolonged orotracheal intubation (≥48 hours) is frequent (2,3). Although information is still limited, patients can experience post-acute consequences, including severe muscle weakness and fatigue, joint stiffness, dysphagia, (neuro)psychological problems, and impaired functioning in terms of mobility and activities of daily living (4). There is, therefore, a need to prepare for the care/rehabilitation of patients in the post-acute phase. This brief communication presents preliminary results on swallowing performance and safety after extubation in patients with COVID-19.

One of the reasons why patients with COVID-19 are considered at risk for dysphagia or for some swallowing impairment is that swallowing and breathing are coordinated functions, *i.e.*, respiration halts when the swallowing reflex is triggered, and they require the activation of common anatomical structures (5). Breathing and swallowing are physiologically linked to ensure effortless gas exchange during oronasal breathing and to prevent aspiration during swallowing. Besides that, prolonged intubation has been shown to alter the mechanoreceptors and chemoreceptors of pharyngeal and laryngeal mucosae, while also causing muscle atrophy and loss of proprioception (6). Reduced pharyngeal and laryngeal sensation places patients at higher risk of silently aspirating food and fluids into the upper airway (*i.e.*, no cough response when aspiration occurs), which can lead to chest infection and pneumonia, malnutrition, increased length of hospital stay, and readmission to the hospital (7).

## Purpose

We investigated the incidence of dysphagia, its time course, and its association with clinically relevant outcomes in extubated critically ill patients with COVID-19. Our results were compared to those in our database system for non-COVID-19 patients who were also subjected to prolonged orotracheal intubation.

## Design

This study was a prospective observational study with systematic dysphagia screening and follow-up until return to safe oral feeding. The project was approved by the Scientific and Ethics Committee of the Institution (CAPPesq HCFMUSP no. 3.992.554). Before their enrollment, all participants were informed of the purpose and procedures of the study, after which they all gave their written informed consent.

## Settings

The study was conducted at the ICU of a tertiary care academic public hospital.

## Patients

Only patients who were referred by the medical team for a bedside swallowing assessment (BSE) were included in the study.

In total, 101 adult ICU patients diagnosed with COVID-19 (median age 53.4±15.9 yr; 66 male and 35 female patients) who were subjected to orotracheal intubation, had a Glasgow Coma Scale score ≥14, and presented with a stable medical respiratory condition according to their medical files were screened for post-extubation dysphagia. Critical ICU patients (CP) from the same institution who were subjected to prolonged orotracheal intubation (≥ 48 hours) served as the confirmatory cohort (n=150; median age 54.0±18.6 yr; 82 male and 68 female patients).

## Interventions

Bedside swallowing evaluation (BSE) included the application of the Dysphagia Risk Evaluation Protocol (DREP) (8), followed by classification of the swallowing functional level according to the American Speech-Language-Hearing Association National Outcome Measurement System (ASHA NOMS) (9). The ASHA NOMS swallowing level scale is a multidimensional tool designed to measure both the required supervision and diet levels by assigning a single number between 1 and 7 (Level 1 - the individual is not able to swallow safely orally. Nutrition and hydration are received through non-oral means; Level 7 - an individual’s ability to eat independently is not limited by the swallow function. Swallowing would be safe and efficient for all consistencies. Compensatory strategies are effectively used when needed). BSE was performed within the first 24 hours after extubation by trained ICU speech-language pathologists. Patients with positive screening were subjected to a confirmatory bedside swallowing examination and follow-up until the return to safe oral feeding.

## Measurements and Main Results

To compare the groups of patients, we also included the following demographic and clinical data: age, sex, presence of comorbidities, orotracheal intubation time (in days), number of swallowing rehabilitation sessions until dysphagia resolution, and swallowing functional level 24 hours after extubation and at ICU discharge. Data were analyzed using IBM SPSS software, version 25. In addition to the descriptive analysis, between-group comparisons were performed using Student’s *T*-test (for quantitative data) or Pearson’s chi-square (for categorical data). The adopted significance level was 5% for all analyses.

Analysis of the demographic and clinical data indicated the following: the groups of patients did not differ in terms of age (*p*=0.089) and sex (*p*=0.092); patients with COVID-19 remained intubated for more days than CP were (8.8±8.1 days and 6.1±3.5 days respectively; *p*=0.002); patients with COVID-19 had higher incidences of neurological disorders (n=3; *p*=0.034), diabetes (n=27; *p*<0.001), and hypertension (n=45; *p*<0.001) than in CP; and CP presented with more pulmonary disorders than in patients with COVID-19 (n=60; *p*<0.001).

The groups of patients differed in terms of the functional level of swallowing 24 hours after extubation (*p*<0.001). On the first swallowing assessment, 19.8% (n=20) of COVID-19 patients presented ASHA levels 1-3 (*i.e.*, the individual is not able to swallow safely orally and an alternative feeding method is required) and 53.5% (n=54) presented ASHA levels 4 and 5 (*i.e.*, swallowing is safe but there are some diet restrictions and cues to use compensatory strategies are required), whereas 40.0% (n=60) of CP presented ASHA levels 1-3 and 26.0% (n=39) presented ASHA levels 4 and 5. Dysphagia resolution also differed significantly between the groups at ICU discharge (*p*=0.005). We observed that 70.3% (n=71) of patients with COVID-19 were able to reach ASHA levels 6-7 (*i.e.*, swallowing is safe, and the individual eats and drinks independently, with requirement of minimal cueing rarely), whereas only 52.0% (n=78) of CP were able to reach the same swallowing functional level.

Another point that significantly differed between the two groups was the number of swallowing rehabilitation sessions until dysphagia resolution. Patients with COVID-19 required 2.9 (±1.7) sessions for dysphagia resolution, whereas CP required 10.5 (±9.3) sessions.

## CONCLUSIONS

Dysphagia after extubation was common in ICU patients with COVID-19 and in CP. However, a greater number of CP sustained dysphagia at ICU discharge. Patients with COVID-19 remained intubated longer and needed fewer swallowing rehabilitation sessions to return to safe oral feeding. These data add to the knowledge on the characteristics of dysphagia in patients with COVID-19 in the acute medical setting. Studies on the underlying causes of why dysphagia does not resolve in some patients are warranted.
